# Telemedicine use in current urologic oncology clinical practice

**DOI:** 10.3389/fsurg.2022.885260

**Published:** 2022-08-19

**Authors:** Nahuel Paesano, Diego M. Carrion, Ana María Autrán Gomez

**Affiliations:** ^1^ Department of Urology, Instituto Médico Tecnológico, Prostate Institute Barcelona, CIMA—SANITAS Hospital, Barcelona, Spain; ^2^ Office of Residents and Young Urologists, Office of Residents and Young Urologists of the American Confederation of Urology (CAU), Barcelona, Spain; ^3^ Department of Urology, Torrejon University Hospital, Madrid, Spain; ^4^ Department of Urology, Instituto de Cirugía Urologica Avanzada (ICUA), Clínica CEMTRO, Madrid, Spain; ^5^ Department of Urology, Lyx Urology, Madrid, Spain; ^6^ Office of Research, Office of Research of the American Confederation of Urology (CAU), Madrid, Spain

**Keywords:** telemedicine, telehealth, urology, COVID-19, videoconsultation

## Abstract

**Introduction:**

In the last fifteen years, there have been important technological advances in telehealth systems and urology, along with other specialties, has been a pioneer in the successful use of this medical care modality. Due to the COVID-19 pandemic, the use of telemedicine has been rapidly embraced by the urology community around the world. A review of the most relevant and updated articles on telemedicine and telehealth in urology has been carried out with the aim of better understanding how it has been implemented to date, as well as reviewing concepts, current regulations, health policies and recommendations for its implementation.

**Methods:**

A narrative review of the current literature published up to April 2022 on the use of telemedicine in urology was performed. From the search results, 42 publications were obtained for analysis.

**Results:**

Telemedicine in urology has been shown to be useful in a variety of clinical scenarios within urological practice, from benign diseases to advanced cancers. This is due to the positive impact on medical consultation times, lower patient mobility costs, less work absenteeism and greater protection for both the patient and the doctor. The main limitations for the use of telemedicine lie in the impossibility of a correct physical examination, which is essential in certain situations, as well as the lack of accessibility to technology in disadvantaged populations and in elderly patients who have not adapted to the use of technology, as well as the lack of development of health policies to establish their implementation in some countries.

**Conclusion:**

Telemedicine is in full growth. There is much information in the current literature on the successful adoption of telemedicine for patients suffering from urological diseases. While the use and implementation of these new practices has been rapid in the urology community, more work is needed to bring more accessible specialty care to underserved and underdeveloped areas. Health policies must promote its development to reduce costs and increase access.

## Introduction

One of the first articles addressing the use of telemedicine in urology was published in 1993 when the establishment of telemedicine was considered a utopia at that time. This article described the results of different video consultations between two military base camps more than 2000 miles apart. The authors concluded that geographical barriers should not be a limitation for diagnosis, treatment, or follow-up of patients (1).

In the last fifteen years, important investments and technological advances have been made in telehealth systems, and urological practice, together with other specialties, has been a pioneer in the successful use of this medical care modality (2, 3). Nowadays, due to the current COVID-19 pandemic, each health system has undergone dynamic changes redirecting its resources to face the pandemic in the best possible way. Telemedicine has been rapidly adopted by the urologic community worldwide with more advantages than disadvantages. Protection measures for health workers and patients have drastically changed healthcare practices worldwide, which is why many centers around the world have implemented telematic systems whose purpose is to reduce unnecessary face-to-face medical visits (4).

While the words “telehealth” and “telemedicine” are often used interchangeably, the former is defined as a tool for remote clinical healthcare, professional education, and public health, while the latter refers more specifically to applications used in the diagnosis and treatment of diseases (5).

Telemedicine can be implemented through different technological modalities including video conferencing software, mobile applications, and portable devices. It can be used to provide direct patients care or it can be used as a means of facilitating professional-to-professional interactions to discuss clinical cases, ask for consultations, or even to discuss cases in multidisciplinary team meetings.

A review of the most relevant and updated articles on telemedicine and telehealth in urology has been carried out with the aim of better understanding how it has been implemented to date, as well as reviewing concepts, current regulations, health policies and recommendations for its implementation.

## Materials and methods

**Figure 1:**
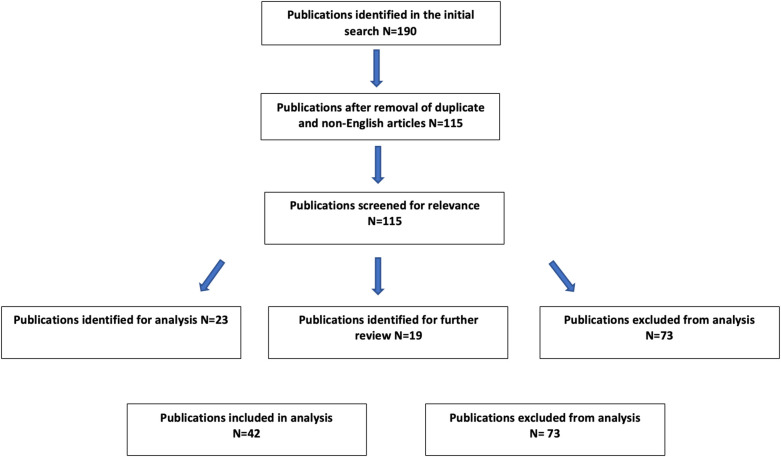
Literature search methodology.

A review of the most relevant and updated articles on telemedicine and telehealth in urology has been carried out by the authors. For this purpose, a biographic search up to April 2022 has been performed in Pubmed and Embase search engines using the following keywords: “COVID-19” AND “Urology” AND (“telemedicine” OR “telehealth” OR “videoconsultation”). All duplicate papers and all non-English language publications were excluded. All study designs and publication types were considered. Each paper was then read by a single reviewer and assigned a score of zero-, one-, or two-based relevance to the topic of telemedicine and telehealth. All papers with a score of zero were removed from the analysis, any paper with a score of two was included in the analysis, and any paper with a score of one was submitted to another reviewer to determine eligibility. Our search methodology is summarized in Figure. 1. We selected 42 publications related to COVID-19, telemedicine, and telehealth in urological practice. General observations and thematic analysis are listed in Table 1. Papers were then analyzed by reviewers and were organized into the following nine sections: telemedicine, telehealth, important benefits of the telemedicine, systems required for the implementation of telemedicine, barriers to the implementation of telemedicine, security restrictions and refund policies, telemedicine in urologic training and recommendations for good practices in the use of telemedicine in urology and telemedicine and health policy. This review is presented in a narrative format.

**Table 1:** Review of the literature.

Authors	Implication to Telemedicine/Telehealth	Observation themes
Ellimoottil et al. (2)	Investigate the use of televisits and teleconsultations for urologic conditions.	Current regulatory and reimbursement policies. Barriers to widespread dissemination and implementation of telemedicine in Urology.
Miller et al. (3)	Review the early experience of telemedicine specifically as it relates to urologic practice and discuss the future implications and the utility of telemedicine as it applies to other fields	Telemedicine services can be implemented through a multitude of modalities, including videoconferencing software, mobile applications, and wearable devices and monitors. Many formats of telemedicine are readily reproducible and relevant to surgical and nonsurgical practices alike, including video visits, online services, electronic consults, and tele-rounding. Barriers currently exist for Medicare reimbursement.
Ohannessian et al (4)	Analyzes the use of telemedicine during the first waves of COVID 19 and its importance in access to health at the worst moment of the pandemic.	For countries without integrated telemedicine within their national health care system, the COVID-19 pandemic is a call to adopt the necessary regulatory changes supporting wide adoption of telemedicine.
Kirshenbaum et al. (6)	Evaluate the socio-economic impact of the use of telemedicine in urology.	The emergence of the COVID-19 public health emergency has propelled telemedicine into the future by alleviating many of the barriers that telehealth adopters faced. With the adoption of telemedicine came socioeconomic disparities in care and access. It is crucial to ensure equal access to this emerging technology.
Chu et al. (7)	Retrospective review examining care delivered through urology telemedicine clinics over a 6-month period.	Ninety-seven unique telemedicine visits were conducted and a total of 171 urologic diseases were assessed. The most common conditions were lower urinary tract symptoms. Patient satisfaction was “very good” to “excellent” in 95% of cases.
Boehm et al. (8)	Prospective structured phone interviews of urological patients (*n* = 399) during COVID-19 pandemic.	Evaluate the suitability for telemedicine, the risk factors, the level of acceptance of the patients and the prevalent pathologies in the consultations.
Boehm et al. (8)	Analyzes urology teleconsultations through the covid 19 pandemic through a prospective study.	Suitability for telemedicine and its risks of COVID-19 were assessed, and readiness for telemedicine and demographic data were collected.
Whitten et al. (9)	Review of different articles on innovation in telemedicine.	It explores the different technologies used, the clinical results, the cost-benefits, the perceptions and the adoption challenges of their use throughout the history of telemedicine.
Castaneda et al. (10)	Evaluate the applications of telemedicine in urology.	Telehealth is sparingly used in urology. Barriers to implementation include technological literacy, reimbursement uncertainties, and resistance to change in workflow.
Pang et al. (11)	Recommendations on smart learning to reduce the impact on the learning curve of residents during COVID-19 pandemic through Telehealth.	The COVID-19 pandemic has had rapid and inevitable effects on health care systems and the training and work plans of urology residents. Smart learning is a valuable strategy for maintaining the learning curve of residents.
Nadama et al. (12)	Evaluate the usefulness of a webinar as a platform to educate students in a United Kingdom clinical academic programme as a telehealth platform.	Demonstrates the usefulness and favorable acceptance of the use of telehealth in students during the pandemic.
Paesano et al. (13)	Evaluate the impact of the covid 19 pandemic on the training of residents of the American Confederation of Urology through a survey.	Despite technological rise of the use of telehealth, 65% respondents affirm their theoretical training has been partially or completely affected.
Novara et al. (14)	Systematic review of the literature on the use of telemedicine in urology.	The available literature indicates that telemedicine has been implemented successfully in several common clinical scenarios.
Dorsey et al. (15)	It reflects on the use of telemedicine and intends to make a projection into the future.	Migration of care from the hospital to the home.
Kahn et al. (16)	Retrospective study combining a systematic listing of ICU telemedicine installations with hospital characteristic data.	Analyze the use of telemedicine in a metropolitan area.
LeRouge et al. (17)	Telemedicine is effective, it encourages self care, and is often preferred over traditional care.	Analyzes telemedicine, health reform, health technology barriers, health technology advancements and legal barriers.
Dubin et al. (18)	Telemedicine usage among urologists during the COVID-19 Pandemic.	The usability of telemedicine and the current barriers to its implementation.
Yang et al. (19)	Telemedicine favors the patient in terms of convenience.	Improved population health.
Shiff et al. (20)	Evaluates patient satisfaction with telemedicine appointments as an alternative to face-to-face appointments in an andrology-focused urology academic practice during the coronavirus disease 2019 pandemic.	Patients were generally satisfied with telemedicine as an alternative to in-person appointments during the coronavirus disease 2019 pandemic. Nonetheless, a substantial portion of patients said they would prefer in person appointments in the future.
Toaff et al. (21)	Evaluates the use of telemedicine in urogynecology and analyzes the level of patient satisfaction.	Clinical judgment and existing data should be used to guide them as to which clinical conditions are appropriate for virtual care.
Margolin et al. (22)	It is a study based on a telemedicine satisfaction survey for both doctors and patients with urological pathologies.	High levels of patient and physician satisfaction for telemedicine visits for management of genitourinary malignancies. Technological barriers were encountered by 9% of patients and were associated with decreased satisfaction.
Yebes et al. (23)	Telemedicine in oncological pathologies.	Tips for its use and optimization.
Ong et al. (24)	To assess the effectiveness of a telemedicine service for ureteric colic patients in reducing the number of unnecessary face-to-face consultations and shortening waiting time for appointments during COVID 19 pandemic.	Around 93.1% of patients reported satisfaction with the service.
Checcucci et al. (25)	Evaluation of the management of benign urological pathology through telemedicine.	Telemedicine approach limits the number of unnecessary accesses to medical facilities. However, infrastructures, health workers and patients should reach out to a computerization process to allow a wider diffusion of more advanced forms of telemedicine, such as televisit.
Nguyen et al. (26)	This is a prospective, non-randomized pilot trial comparing telephone consultations (TC) versus video consultations (VC) in urology outpatient clinics.	Patients’ satisfaction was greater with VC compared to TC. Both modalities were associated with many cost benefits for patients.
Socarrás et al. (27)	Recommendations of the European Association of Urology on the use of telemedicine.	The advantages of using telemedicine for patients are recognized, but recommendations for its correct use are detailed.
Lawrentschuk et al. (28)	Use of telemedicine and telehealth in continuous urological training during the covid 19 pandemic.	A positive previous experience. Efficacy of modality.
Khusid et al. (29)	This article details the role of urology residents in the face of the covid pandemic.	The importance of telemedicine in patient care and telehealth in their training.
Tabakin et al. (30)	This article discusses in detail the sudden change that occurred during the pandemic in the training of urology residents.	From this situation, virtual education programs have been developed that can become a national video based curriculum for urology residents, incorporating both didactics and training in surgical skills.
Sen et al. (31)	From the use of telehealth, an E-learning education model for urology residents was created.	This is reliable and easily accessible e-learning platform for the standardisation of training in urology.
Khusid et al. (32)	Use of telemedicine by a urology service in the United States during the COVID 19 pandemic.	Improved population health.
Naik et al. (33)	This study aims to understand the behavioral attitude and perceptions of the population regarding telemedicine and, in doing so, make the services more user-friendly for patients.	Of the total respondents, 35.3% of patients never encountered telemedicine before and 26.9% did not come across telemedicine even during the COVID-19 pandemic.
Gadzinski et al. (34)	This article states that Telehealth in urology is possible to continue after the COVID-19 Pandemic.	Benefit of telemedicine so as not to overload the health system and attend to pathologies that can be resolved by this modality.
Almannie et al. (35)	During the COVID-19 pandemic, most urologists adopted telemedicine technology rapidly.	The limitations of telemedicine should be respected in order to avoid compromising patient safety.
Naik et al. (36)	This review focuses on identifying the outcomes of the recent studies related to the usage of video consulting in urology centers for hematuria referrals and follow-up appointments for a variety of illnesses.	Telemedicine has proven beneficial in such patients and is a reliable, cost-effective patient-care tool, and it has been successfully implemented in various healthcare settings and specialties.
Ayoub et al. (37)	Telemedicine and Telementoring in Urology.	Despite the multiple benefits of telemedicine in urological practice and telehealth in continuing medical education, there are still several barriers that prevent a full integration of telemedicine, such as cost, ethical considerations, security, bandwidth, latency and licensing difficulties.
Bokolo et al. (38)	This article provides a flowchart for synchronous or asynchronous telemedicine patient care.	Care enabled through smart mobile technology. A positive previous experience. Efficacy of modality Technical literacy Improved population health
Malvey et al. (39)	Policy should verify efficacy of each application but reduce barriers to approval to encourage innovation. Due to proliferation of smart mobile devices, telemedicine services should extend to this modality.	Improved interoperability Increased access. Misaligned incentives. Can raise ethical issues.
Kruse et al. (40)	It carries out an exhaustive review of the literature about health policies and telemedicine.	It details the effectiveness and costs of telemedicine in public health and reports on barriers and strengths.
Lee et al. (41)	Policy in developing countries can focus on telehealth first because a cellular network is usually present.	Reaches developing countries Socioeconomics Care enables other resources (educational/technological)
Saleh et al. (42)	Health policy in telemedicine must include displaced populations such as refugees.	Care enables other resources (educational/technological) Increase health outcomes Increased access Reaches developing countries
Griffiths et al. (43)	There are many advantages to the use of telemedicine, and it encourages self-care. There are concerns over data security, patient safety, and the increased cost to the provider for start-up and maintenance costs.	Improved population health Self-efficacy Can raise ethical issues Data security Patient safety Increased cost (to provider) because reimbursement does not cover the technology Increased patient-to-provider communication

## Results

The terms “telemedicine” and “telehealth” are often used interchangeably in the current literature. However, the term “telemedicine” predates “telehealth” in the literature. Telehealth reflects a more recent and comprehensive idea beyond medical care. We can think of telehealth as information and communication technologies that improve health in general and that encompass all aspects of medical care and continuing medical education, while telemedicine refers specifically to technologies used for the diagnosis and treatment of diseases (2, 6).

### Telemedicine

There are different modalities of how to apply telemedicine effectively, always keeping in mind the final objective that will be to provide a quality medical consultation at a distance. The most used format is video consultation, and it consists of a live face-to-face electronic audiovisual interaction between the physician and patients. Despite the limitations of not being able to perform a proper physical examination, video visits have proven to be a reasonable alternative to traditional in-person visits (3).

In a survey published by Chu et al., of more than 1000 patients in the United States, 95% rated satisfaction with video consultations from very good to excellent, and 80% of urologists rated the encounters as excellent. Among the advantages that patients list for this type of modality are: equal consultation times, less waiting time, reduced time off work for the patient, and no travel expenses (7).

In another study done by Bohem et al. 84.7% of interviewed patients wished for a telemedicine consultation in urology rather than a face-to-face consultation, while physicians considered that most patients (63.2%) of their regular clinical practice were judged suitable for telemedicine (8).

Other possible methods for teleconsultation include email, text messages, and other specific software platforms for instant messaging. In the case of older patients, less used to modern technologies and software, the use of telephone calls is a valid and very accepted practice that allows fluent communication between the health care professional and the patient (44).

### Telehealth

There are three main types of telehealth applications: synchronous, asynchronous or store forward, and remote patient monitoring (9, 10). Synchronous telehealth applications (in real-time) are carried out through remote visits. Asynchronous applications involve the collection and storage of health information for later review, these include electronic consultations and communication with patients through a health portal. Remote patient monitoring is a type of asynchronous telehealth that involves the regular collection of patient health data, such as vital signs, and transmission to a provider for monitoring or response. Finally, telehealth can provide education and training activities for residents and urologists (telementoring), and surgical procedures can be transmitted live to distant audiences. In recent years, urology training has undergone gradual modifications according to the escalation of the alter level in each country for COVID-19 (11, 34). In general, interhospital training instances were suspended, residency admission exams were delayed and face-to-face academic activities were stopped, initiating a new stage in scientific dissemination where software like Zoom Meeting, Skype, Webex Cisco, among others, play a fundamental role (11, 28, 44, 45). In the article by Claps et al., the residents provided a perspective on the potential educational value of smart learning, defined as any modality of teaching activity carried out through virtual platforms or online communication channels. The acceptance and impact on the training of residents was considered positive in each of the educational platforms (46). In a survey developed by Amparone et al. on 351 residents, the negative impact on both clinical and surgical activities was evidenced, highlighting the importance of telehealth with virtual training platforms as a fundamental ally in the training of residents in the pandemic and the first years of the post pandemic by COVID-19 (47).

The new virtual courses and lectures produced during the pandemic allowed urology residents to tailor courses to their interests (29). These courses prompted discussion of a shift towards standardized virtual-based curriculums for programs (30, 31). Though virtual didactics may facilitate ease of attendance, that does not always translate into increased participation. Several virtual sessions per day may lead to fatigue, and the ability to attend didactics remotely has created an expectation that residents attend all meetings, regardless of their location or scheduled.

### Important benefits of telemedicine

The current literature indicates that telemedicine in urology has proven to be useful in a variety of clinical scenarios within a urological practice, from benign disease to advance cancers, and from the initial diagnosis to follow-up. In the review published by Novara et al., clinical scenarios ranging from decision-making process to follow-up in prostate cancer, uncomplicated urinary stones, and urinary incontinence have enough evidence in the literature to be highly recommended to be managed safely and efficiently by teleconsultations (14).

Telemedicine not only complies with the current demand of social distancing in order to avoid possible outbreaks in the hospital waiting rooms, public transportation or other places related to health care providers and patients' mobilization. In some rural and underserved areas, with notable workforce shortages, specialized teleconsultations play an important role, improving patients access to care in a more time efficient, safer way (6, 44). Most of the regular follow-up visits of chronic, well-controlled disease can be safely managed with a short video or telephone consultation in which the physician or nurse has access to laboratory or imaging tests (44).

Most of the initial limitations of this practice have been removed and allow personal and patients to gain experience and become used to schedule video consultations or telephone consultations. Telemedicine is yet to be widely accepted in the urological community due to several limitations pertaining to patient and physician acceptance, licensure and liability, costs, safety, ethical considerations, and changes in workflow (37).

Some health care authorities and professionals worried initially that telemedicine technologies will result in an unequal distribution of health care resources, as telehealth companies are focused on providing software for well-resourced patients in order to expand their presence. But the truth is that in low income communities, some easier and less sophisticated methods of telemedicine such as telephone calls, can help these population to get access to specialized care (6, 38).

### Systems required for the implementation of telemedicine

Telemedicine activities should be carried out using a secure and robust internet-based network, which generally involves the use of a virtual private network combined with end-to-end encryption software that meets recognized standards. The goal should be to transmit and store data securely at all times. There are several internet-based video conferencing software programs that are commercially available for synchronous applications, but the electronic medical record is all that is required for asynchronous applications. Currently, there are commercially available platforms like EPIC ® medical record system and NHS Attend Anywhere ®. But Zoom, Doxy.me, WhatsApp, and Skype are also used with great frequency. Technological failures can cause interruptions during the practice of teleconsultation, and therefore a plan should be ready in this case, health care providers and patients should have contact information for technical support at all times for case troubleshooting (3, 48).

### Barriers to the implementation of telemedicine

At the patient level, there are some important possible barriers in the practice of telemedicine. While telehealth is intended to greatly improve patient access to medical care, access to technology remains a limiting factor. In older patients, there can be troubles in adopting the technology and devices needed for telemedicine (15, 49).

On the other hand, difficulties have also been identified in implementing telemedicine at the provider level. While most physicians are familiar with telemedicine, many report limited experience with its use. Carrying out telehealth, particularly those modalities that use newer technologies, requires more than medical training (16, 17).

The lack of experience in teleworking and telehealth could precipitate challenges for health care providers, which include scheduling teleconsultations, attending remote team meetings, maintaining self-discipline, keeping away distractions, avoiding feelings of loneliness, and creating a professional work environment (50).

In a survey developed by Dubin et al., 620 urologists from 58 countries on five different continents showed that approximately half of the urologists surveyed have never used telemedicine in their life. When addressing the key barriers to telemedicine use for those that had experienced, the top three reasons were: patients' lack of technological understanding, patients' lack of access to the required technology, and concerns about reimbursement. Another major barrier found was the lack of administrative support, which was the fourth most mentioned barrier to the use of telemedicine (18).

Another interesting point against telemedicine, from another study, was that some clinicians felt that increased reliance on telehealth could disrupt the patient-provider relationships (10).

### Security restrictions and refund policies

Adherence to security policies for patient data is essential, as is carried out in face-to-face visits and telemedicine support programs, in any of their modalities should guarantee them.

The differences in the regulations of each country directly impact the reimbursement models for telehealth services, providing a significant barrier to their implementation. Collective strategies vary even in the same country (10). A common restriction is on the applicable technology. Since many countries or states specifically restrict their definition of telemedicine to real-time technologies, asynchronous technologies and remote patient monitoring are rarely reimbursed. Similarly, email, fax, and telephone, while frequently used to provide continuing medical care, are rarely accepted forms of telemedicine for reimbursement (19).

### Telemedicine in urologic oncology

Patients with urological malignancies have a greater number of risk factors for a severe course of COVID-19 than patients with non-oncological diseases (8). The emergence of the pandemic resulted in rapid and widespread adoption of telemedicine. Urologists quickly adopted telemedicine to facilitate social distancing, continue to care for their patients and keep practices economically viable. During the annual 2020 AUA census, as much as 71.5% of urologists reported engaging using these practices during the COVID public health emergency (32, 51).

Thus, in most of the published studies, there is evidence of high assimilation of telemedicine by both patients and professionals. There are publications related to the use of telemedicine in the different subspecialties of urology, such as pediatric urology, andrology, urogynecology, and oncology. Although the main limitation is the impossibility of performing a physical examination, all the studies evaluated in this review highlight the high rate of patient and professional satisfaction (20–22, 33, 52).

Given its proper use and always being aware of its limitations, telemedicine has an opportunity as a safe and efficient alternative for the management of uro-oncological patients in many situations. Health care in this setting should be personalized according to patient and disease characteristics as much as possible and an initial basic triage is mandatory to identify those patients who require to go to the hospital for a physical examination, procedure, or admission to the emergency department (23). Although the use of telemedicine in urology has been successfully implemented even in acute management pathologies such as ureteral colic, reducing face-to-face consultations during the pandemic (24).

As explained before, the key limitation is adequate access to technology, especially in the older patients. This disparity in technology is underlined by a recent study in Italy showing that more than half of patients contacted regarding benign urological conditions did not have access to the technology required for a telemedicine visit. As telemedicine continues to grow, it needs to do so while addressing the needs of vulnerable groups (25, 35). Telemedicine is better suited for long-term follow-up as well as reports on chronic illnesses (36).

Regarding the modality, one study compared patient satisfaction between teleconsultation (TC) and videoconsultation (VC). Forty-eight urology patients who were managed by TC and 66 VC patients were included. The differences between the two groups of patients were small but tended to favor VC. Patient satisfaction was higher with VC compared to TC. Both modalities were associated with many cost benefits for patients (26).

### Recommendations for good practices in the use of telemedicine in urology

The European Association of Urology (EAU) published a series of recommendations in order to promote best practices in telemedicine use (27).

Here we provide a summary of the key recommendations:

- Stay up to date on innovative strategies and learn to use platforms and tools that enable communication with patients, communication with team members, and secure data exchange.
- Provide patients with different methods of scheduling appointments. Contact patients in advance to agree on the consultation time and provide them with basic instructions on how to access the required software if used. Provide a telephone number for urgent inquires and alert symptoms and avoid unnecessary visits to the hospital.
- During video consultations try to have a quiet and private environment and make sure the patient has it too. Preferably, the patient should be alone or with a family member to help him with technical issues. Computers are preferable to mobile phones for video consultation and cameras should be placed at eye level. Try to wear professional “work” clothes. Manage body language and analyze the patient's body language. Offer tips for conducting guided and focused self-physical examinations if necessary. For patients who cannot set up a video visit for whatever reason, phone calls may be the best alternative.
- Not having a specific application or software is not a valid excuse for avoiding telemedicine. Even phone calls and access to medical records managed by a college or other health care provider can help address urological consultations during a pandemic emergency.
- Hospital phones should be used for phone calls to patients. If you want to use your personal mobile phone, it is better to configure it in such a way that it does not display your personal number. Corporative hospital email should be used as much as possible in telehealth, avoid using personal email accounts which can compromise patients' information.
- Patients should be triaged using common and clinical sense.
- Submit reports, send prescriptions, and schedule laboratory or diagnostic tests with the support of an administrative team.
- Maintain constant communication within your team.
- Become familiar with the available options for corporative email, video conferencing, calendars, social media, telehealth platform packages, and access to webinar platforms.
- Comply with the privacy and billing regulations of your country and region.
- If you are a healthcare provider, self-discipline is crucial. Set hours and avoid distractions when you are working from home. Create healthy routines and keep your motivation high.
- Stay up-to-date academically by following the virtual congresses, webinars, guidelines, and articles from the official channels of the key national and international urological associations.
- Participate and organize scientific update meetings by videoconference. Discuss relevant clinical cases and new strategies with novel situations.
- Try to generate and share quality content for the population and patients. Remember that you are a healthcare professional and there is a substantial need to disseminate high-quality healthcare information, especially during a public health world crisis.

### Telemedicine and health policy

The spread of telemedicine is reliant on health policy as it would allow and encourage the use of telemedicine to improve the cost, quality, and access (39). The characteristics of telemedicine are clearly in favor of the patient in terms of comfort and privacy; however, the reimbursement and incentives do not align with this modality (40).

You want it then you get it; important computer systems are not always necessary when developing telemedicine protocols to facilitate public health access to the entire population. Interventions of the Simple Messaging System (SMS) can occur even without data service. A simple protocol has been carried out and has shown great promise in developing countries (41). Telemedicine allows providers to reach remote areas of developing countries where broadband is not strong, but a cellular network exists. This protocol does not require a data network, but instead transmits small message packets over a cellular network (42). But the telemedicine modality is not free. There are significant costs up front and some basic training for both vendors and staff to use the technology safely and effectively. Ongoing costs are typically negligible, certainly less than the incremental cost associated with expanding clinic space. The initial costs for patients are almost negligible if the patient already has the technology with which he will be able to access the telemedicine system (43).

Telemedicine continues to increase in prevalence around the world but there are barriers to the adoption of telemedicine and its correlation with health policy. The barriers consist of current health policy that limits providers within states, the cost of implementation, ethical concerns, limited resources, the digital age divide, and the current reimbursement model. As this new modality of care becomes more widely accepted and preferred, nations health policy will need to adjust and expand to govern and monitor it while incentivizing providers and patients to use it (40).

The present study is not devoid of limitations. Although this review was carried out in detail and the available works were of good methodological quality, since it is a narrative review, the selection of articles may be biased according to the criteria of each of the authors when including or excluding articles for this review.

## Conclusion

Telemedicine is in full growth. There is increasing information in the current literature on the successful adoption of telemedicine for patients suffering from urological diseases. The approval and satisfaction of patients and doctors with telehealth is a fact. This is due to the positive impact on medical consultation times, lower patient mobility costs, less work absenteeism and greater protection for both the patient and the doctor. The main limitations are the lack of physical examination and limitations in technology availability or appropriate use for patients. Although the use and implementation of these new practices has been rapid in the urology community, more work is needed to bring more accessible specialty care to underserved and underdeveloped areas. Health policies must promote its development to reduce costs and increase access. More research articles can help uncover just how beneficial and affordable telemedicine can be.

## Data Availability

The original contributions presented in the study are included in the article/**Supplementary Material**, further inquiries can be directed to the corresponding author/s.
